# Tuberculosis

**Published:** 1891-07

**Authors:** 


### TUBERCULOSIS.

The following paragraph was ommitted from Dr. Lee's article on the "Prevalence of Tuberculosis" in the June number of the JOURNAL, coming after the paragraph beginning "The use of Koch's lymph," etc.

I had two cows at the Harvard Veterinary Hospital, through the courtesy of Dr. Lyman, on which to try the action of Koch's lymph, which Dr. Stone, of Boston, was going to procure directly from Dr. Koch.

One of these cows died and the other was killed before the arrival of the lymph.

Dr. Stone thought so little of its value as a diagnostic agent, from experiments on people, that when the lymph finally did arrive he did not think it worth while for me to get any other cases for experiment.

